# A Novel Method for the Pre-Column Derivatization of Saccharides from *Polygonatum cyrtonema* Hua. by Integrating Lambert–Beer Law and Response Surface Methodology

**DOI:** 10.3390/molecules28052186

**Published:** 2023-02-26

**Authors:** Hui Liu, Yuanyuan Zhao, Leijing Chen, Jiao Du, Hongyan Guo, Bin Wang

**Affiliations:** 1 Key Laboratory of Xin’an Medicine, Ministry of Education, Anhui University of Chinese Medicine, Hefei 230038, China; 2 Hefei Institutes of Physical Science, Chinese Academy of Sciences, Hefei 230031, China; 3 Institute of Pharmaceutical Chemistry, Anhui Academy of Chinese Medicine, Hefei 230038, China

**Keywords:** quality control, chemosensor, pre-column derivatization, *P. cyrtonema* Hua. polysaccharides

## Abstract

Traditional Chinese medicine (TCM) safety and effectiveness can be ensured by establishing a suitable quality assessment system. This work aims to develop a pre-column derivatization HPLC method for *Polygonatum cyrtonema* Hua. quality control. In this study, 1-(4′-cyanophenyl)-3-methyl-5-pyrazolone (CPMP) was synthesized and reacted with monosaccharides derived from P. cyrtonema polysaccharides (PCPs), followed by HPLC separation. According to the Lambert–Beer law, CPMP has the highest molar extinction coefficient of all synthetic chemosensors. A satisfactory separation effect was obtained under a detection wavelength of 278 nm using a carbon-8 column and gradient elution over 14 min, with a flow rate of 1 mL per minute. Glucose (Glc), galactose (Gal), and mannose (Man) make up the majority of the monosaccharide components in PCPs, and their molar ratios are 1.73:0.58:1. The confirmed HPLC method has outstanding precision and accuracy, establishing a quality control method for PCPs. Additionally, the CPMP showed a visual improvement from colorless to orange after the detection of reducing sugars, allowing for further visual analysis.

## 1. Introduction

Chemists have frequently shown a significant interest in the design and synthesis of novel chemosensors to determine the analyte in vivo and in vitro [1,2,3]. As a significant pharmacological component in TCM, polysaccharides have strong biological effects, such as immunological modulation and tumor prevention [4,5]. To ensure clinical safety and efficacy, the quality control of TCM polysaccharides is crucial. The polysaccharides’ inherent polydispersity and lack of chromophores make polysaccharide determination difficult [6,7], not to mention the difficulties of establishing a consistent method of quality control for Chinese polysaccharide medicine. 

Saccharides are extremely difficult to detect due to their strong polarity, structural similarity, and lack of UV absorption or luminescence group. Generally, the number of total saccharides in crude polysaccharide extracts is determined by colorimetric methods after acidification, based on the Chinese Pharmacopoeia [8]. However, the direct test’s sensitivity and accuracy are low [9]. By comparison, colorimetric chemosensors have received increased attention due to their excellent sensitivity and operational simplicity [10,11,12]. 

Chemical derivatization can significantly improve the measured sensitivity and selectivity of saccharides in a derivatization reaction between the chemosensor and saccharide, particularly in pre-column derivatization, which requires only a few diverse derivatization reagents, such as 1-phenyl-3-methyl-5-pyrazolone (PMP) [13], fluorescein isothiocyanate (FITC) [14], and others [15,16]. In 1989, Honda et al. [17] used PMP to derivatize monosaccharides, and subsequently, researchers used the HPLC-UV technique to examine the PMP derivatives, achieving a desirable result. It should be noted that PMP is currently the pre-column derivatization chemosensor with the highest usage rate. However, the stronger alkaline conditions and lower detecting wavelength result in drawbacks such as undesired byproducts, contaminant interference, and excessively high sensor requirements. As a result, a chemosensor that has a higher sensitivity, lower sensor requirements, and mild reaction conditions is highly desired.

Following our research interests in chemosensor design and quality control [18,19,20,21], particularly for *Polygonatum* by the HPLC technique [16], herein, we designed and synthesized a variety of PMP derivatives comprising diverse functionalization groups, such as methyl, methoxy, halogen, cyano, and nitro, to address the difficulties stated above. Combining the Lambert–Beer law and response surface methodology (RSM) [22,23], the CPMP with the highest molar extinction coefficient was screened out. By utilizing the RSM combined with the HPLC technique, we established a method for the visual sensing of monosaccharides from PCP. Finally, we established a consistent method of quality control for *P*. *cyrtonema* Hua., highlighting the value of the novel technology. 

**Scheme 1:**
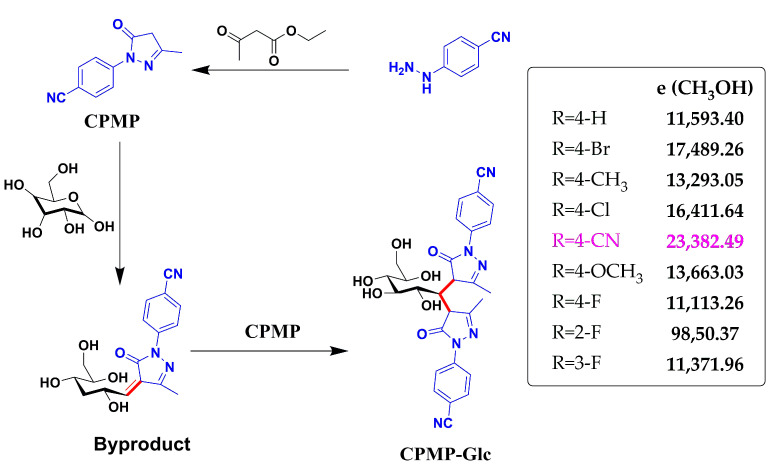
The workflow of sensing monosaccharides through a PMP-based chemosensor. (Blue: chemosensor; Black: material and analyte; Red: new bond; Purple: optimal group and ε value).

Some efficient and simple reagents for derivatization have been reported [24,25]. In this study, the workflow of sensing monosaccharides was depicted in Scheme 1. The molar extinction coefficient of CPMP is 23382, while that of PMP is 11593, indicating that CPMP is twice as sensitive as PMP. Meanwhile, the maximum absorption wavelength of CPMP is 278 nm, whereas the maximum absorption wavelength of PMP is 245 nm, which indicates less noise interference when CPMP is used. For the developed approach, the limit of detection (LOD) is less than 0.006 μg/mL in terms of the detection of monosaccharides. In contrast, among the other reported approaches [26,27,28], the LOD is greater than 0.4 μg/mL. Consequently, the approach developed in this study offers significant advantages.

## 2. Results

### 2.1. UV-Vis Spectra of CPMP 

**Figure 1:**
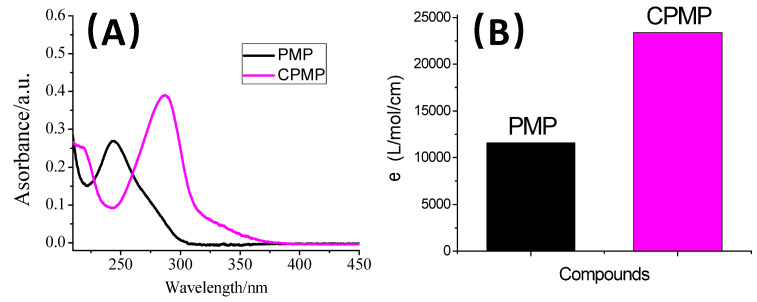
Spectroscopic properties of pre-column derivatization reagents of PMP and CPMP. (**A**) UV-Vis spectrum; (**B**) Molar extinction coefficient in methanol.

A UV-visible spectrophotometer was utilized to determine the maximum absorption wavelength of the synthesized CPMP. Lambert–Beer’s law was used to calculate the molar extinction coefficient. The PMP molecule exhibits an ultraviolet absorption peak of 245 nm, whereas CPMP exhibits an absorption peak of 278 nm, which is higher in magnitude than PMP (Figure 1A). A comparison of the molar absorption coefficient of PMP (ε = 11593.40 L/mol/cm) and CPMP (ε = 23382.49 L/mol/cm) for CH_3_OH indicates that CPMP is more sensitive than PMP (Table 1, and Figure 1B). Recently, we have developed a pre-column derivatization HPLC method based on the reaction of 4-hydrazine-1,8-naphthalimide (HAN) as a new chemical sensor with reducing sugar. In the molecule of HAN, the molar extinction coefficient in methanol is 16138.51 L/mol/cm [16]. In contrast to PMP, the synthesized CPMP has a higher sensitivity.

### 2.2. Optimization of Derivatization Conditions by RSM

After performing a single-factor analysis, we used triethyl amine as the best type of alkali from DMAP, Na_2_CO_3_, NaOH, K_3_PO_4_, and (C_2_H_5_)_3_N. Subsequently, it was determined that reaction temperature, reaction time, alkali concentration, and CPMP concentration played a significant role. The test was designed by the Box–Behnken center combination using Design-Expert 8.0.6 software. Table S1 illustrates the results of the test factors: the peak area of monosaccharide-CPMP varied from 66.3 to 73.45. As the objective function of the regression equation, we obtained the quadratic equation. Subsequently, using the F-test and *p*-value, it is possible to evaluate the significance of the model’s coefficients. The data obtained from the experiment are fitted multiple times to generate a mathematical model:

Y = 71.55 + 0.4667A + 1.39B − 0.0917C − 1.98D = 0.0750AB + 0.2250AC + 0.0000AD + 0.2250BC − 0.3750BD + 0.3250CD − 1.44A2 − 1.33B2 − 1.25C2 − 0.7645D2

where Y stands for the peak area of the Glc-PMP derivative, while A, B, C, and D represent reaction temperature, reaction time, alkali concentration, and CPMP concentration, respectively. The significant influencing factors were B (Time, *p* < 0.0001) and D (CPMP concentration, *p* < 0.0001), as shown in Table 2.

As a result of these data, it is feasible to establish a model through experimentation. As can be seen from the precision value of 10.655, the model is suitable for forecasting the outcomes of experiments. With an R^2^ adjusted value of 0.8047, the model can predict a response value of 80.47%. With a determination coefficient of R^2^ of 0.9023, the model has excellent suitability and can be used to analyze and predict the peak area of monosaccharide-CPMP. With the R^2^_Pred_ equal to 0.7327, there is no significant difference between it and the R^2^, indicating that it was unnecessary to investigate further.

**Figure 2:**
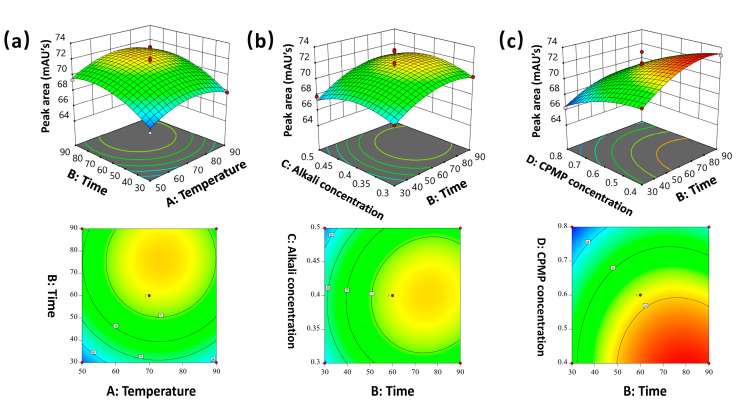
Response surface plots showing the effects of variables on the derivatization of CPMP. (**a**) The response surface of the impact of exaction temperature (A, °C ) and time (B, min ); (**b**) The response surface of the effects of temperature (B, min) and alkali concentration (C, mol/L); (**c**) The response surface of the impact of time (B, min) and CPMP concentration (D, mol/L). Red means the effect is the greatest; Yellow means the effect is the moderate, and the Green is the weakest.

Our study used Design-Expert (11.0) to establish the relationship between the independent and dependent variables. Figure 2 depicts the independent and dependent variables’ 3D response surface and contour plot and presents the response surface and contour map of A (Temperature) and B (Time) about the peak area of monosaccharide-CPMP. Based on the map’s intensity of contours and the response surface’s steepness, the map reflects how many interlacing factors influence the response surface. With increasing density and slope, the impact degree will be more pronounced. Maps in Figure 2b is the steepest (corresponding to Figure 2a–c). These results illustrated that time (B) and CPMP concentration (D) significantly influenced the peak area of monosaccharide-CPMP, while temperature (A) and alkali concentration (C) had a slight influence. Overall, the pre-column derivatization conditions of monosaccharide-CPMP were optimized using RSM during the experiment. As a result, the optimal conditions were established below: time: 60 min, temperature: 70 °C, alkali concentration: 0.4 mol/L, and CPMP concentration: 0.6 mol/L.

### 2.3. Mechanism Analysis of CPMP-Glc by UV-Vis and HRMS

**Figure 3:**
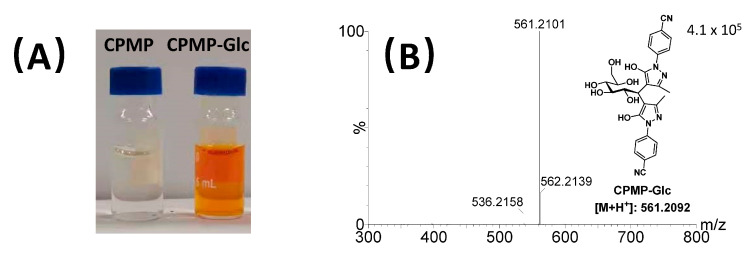
Mechanism analysis: (**A**) Photograph of CPMP and CPMP-Glc solutions; (**B**) HRMS data of CPMP-Glc.

UV-Vis, HRMS, and NMR techniques were used to identify the mechanisms involved in derivatization [29]. As shown in Figure 1A, the maximum absorption wavelength of CPMP is 278 nm, which is redshifted compared with that of PMP. Figure 1B shows that CPMP is attached to the benzene ring with substituent 4-CN, increasing its molar extinction coefficient. In Figure 3A, the methanol solution of CPMP is colorless and transparent, while the solution of CPMP-Glc is orange, suggesting the absorption spectrum has changed. Finally, HRMS spectra provide support for the structure of CPMP-Glc. The peak at *m*/*z* 561.2101, as shown in Figure 3B, was assigned to [2CPMP-Glc + H]^+^ (calc. *m*/*z* 561.2092).

### 2.4. Quality Control for P. cyrtonema Hua.

When monosaccharides are analyzed directly by HPLC-DAD, they cannot be observed or detected due to the lack of ultraviolet chromophores in saccharides. However, the derivatization reaction between chemosensor CPMP and three monosaccharides, Glc, Man, and Gal, allows the observation of the metabolites of the various monosaccharides conjugated with CPMP. These metabolites are easily separated by the established method. 

**Figure 4:**
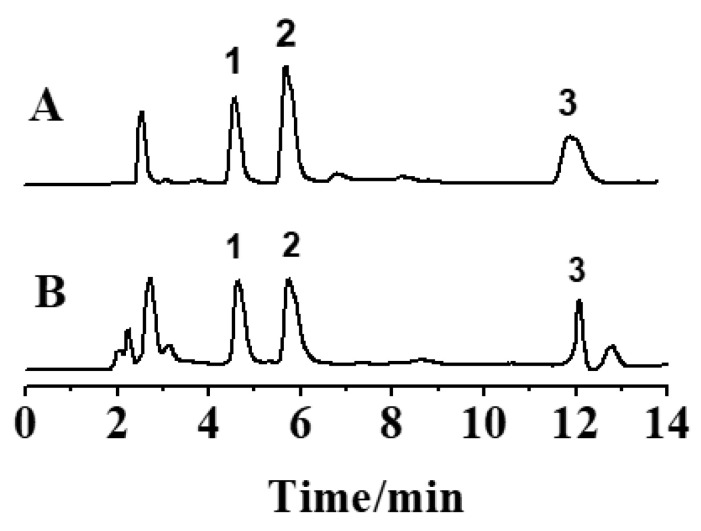
(**A**) HPLC-DAD chromatogram of mixing CPMP-monosaccharide standards; (**B**) HPLC-DAD chromatogram of PCPs after derivatization; (peak 1, CPMP-Gla, *t*_R_ = 4.78 min; peak 2, CPMP-Glc, *t*_R_ = 5.92 min; peak 3, CPMP-Man, *t*_R_ = 12.03 min).

A comparison of the results displayed in Figure 4A may be sufficient to satisfy the requirements of qualitative and quantitative analysis. Afterwards, an indirect study of monosaccharides in PCPs samples was conducted. The first step involved the hydrolysis of PCPs into monosaccharides with 2 M trifluoroacetic acid, followed by the derivation of the monosaccharides with CPMP. Using the HPLC technique, the obtained samples were analyzed. The HPLC chromatogram shown in Figure 4B exhibited three peaks identified and labeled as follows: peak 1, CPMP-Gal, *t*_R_: 4.78 min; peak 2, CPMP-Glc, *t*_R_: 5.92 min; and peak 3, CPMP-Man, *t*_R_: 12.03 min. A separation degree more significant than 1.5 has been achieved between the three components above-mentioned, thus meeting the requirements for HPLC separation. Therefore, this established method is a practical approach to separating CPMP-monosaccharides and determining indirect monosaccharide content. On the basis of the linear equation, the contents of CPMP-Gal, CPMP-Glc, and CPMP-Man were calculated. As displayed in Table 3, the corresponding molar ratio of Gal, Glc, and Man is 0.58:1.73:1.00 in PCP samples. The glucose content was the highest, consistent with literature reports [30,31]. Generally, we were able to successfully detect monosaccharide composition in PCP analytes using the established method, satisfying the goal of providing a method for the quality control of *P. cyrtonema* Hua. polysaccharide. 

### 2.5. Method Validation

To validate the developed method, we conducted experiments examining linearity, LOD, and reproducibility. After plotting the relationship between peak area and spiked concentrations of Glc, Man, and Gal, the linear fitting to the results was completed. Calibration curves are constructed using Glc, Man, and Gal standards with a concentration range of 1 to 104 μg/mL. The linear regression and LOD results are displayed in Table 4. A satisfactory correlation coefficient ranged between 0.9993 and 0.9997 for the three compounds. LOD values were determined for the three substances and ranged from 2.12 × 10^−3^ to 5.66 × 10^−3^ μg/mL. In addition, a six-time continuous injection was used to evaluate the precision of the present method, which laid the foundation for calculating the relative standard deviation (RSD) for CPMP-Glc. The RSDs were 0.44% (Table 5). The stability of the established method was also measured during the 24 h after preparation. The RSD of the stability was 0.31%, exhibiting that the reproducibility of the presented method is excellent. As a result of its excellent linear relationship, accuracy, sensitivity, and stability, the validated method is able to satisfy the requirements of determining monosaccharides.

## 3. Materials and Methods

### 3.1. Reagent and Instrument

We collected the rhizomes of *P. cyrtonema* Hua. (PC) identified by Dr. Jinmei Ou, Anhui University of Chinese Medicine, from Banzhuyuan in Jinzhai County (Luan, Anhui Province, China). 

The purity of the 4-phenylhydrazine hydrochloride acquired from Shanghai Macklin Biochemical Co., Ltd. (Shanghai, China) was 95%, and it has various substituents (4-Br, 4-CH_3_, 4-Cl, 4-CN, 4-OCH_3_, 4-F, 2-F, and 3-F). Shanghai Bide Medical Technology Co., Ltd. (Shanghai, China) supplied the monosaccharide standards, including glucose (Glc), mannose (Man), and galactose (Gal) (purity > 97%). Aladdin Reagent Co., Ltd. (Shanghai, China) provided 1-phenyl-3-methyl-5-pyrazolone (PMP, purity 99%); reducing sugar-CPMP derivatives were separated using an Agilent ZORBAX SB C8 column (4.6 mm × 250 mm, 5 μm particle); MERCK & Co., Inc. (Shanghai, China) provided the chromatographic methanol and acetonitrile; Waters Xevo G2-XS QTOF spectrometers were used to collect the high-resolution mass spectra (HRMS) (Tolerance = 10.0 ppm). An ultraviolet–visible spectrophotometer, SHIMADZU UV-2550, was used to measure the absorption of ultraviolet-visible light.

### 3.2. Synthesis of CPMP

4-(5-hydroxy-3-methyl-1H-pyrazole-1-yl) benzonitrile **3e** was synthesized according to a reported procedure [32,33,34]. In brief, ethyl acetoacetate (2 mmol, 252 μL), glacial acetic acid (0.6 mL), and ethanol (2 mL) were added to commercially available 4-cyanophenylhydrazine hydrochloride (2 mmol, 306.28 mg), and the mixture was reacted at 100 °C for 6 h. A light-yellow solid powder was obtained by silica gel column chromatography, with a yield of 10%. NMR analysis of the prepared compound showed that the compound was 4-(5-hydroxy-3-methyl-1H-pyrazole-1-yl) benzonitrile (CPMP). ^1^H NMR (500 MHz, DMSO) δ 7.98 (d, 2H), 7.88 (d, 2H), 2.51 (s, 2H), 2.14 (s, 3H). This compound was known [31]. Later, we carried out substrate expansion. PMP derivatives with different substituents (such as 4-H, 4-Br, 4-CH_3_, 4-Cl, 4-OCH_3_, 4-F, 3-F, and 2-F) were synthesized according to the method described above. These compounds were known [32,33,34].

### 3.3. Investigation of Spectroscopic Characteristics

A total of 20 mg of each PMP derivative with different substituents (4-H, 4-Br, 4-CH_3_, 4-Cl, 4-CN, 4-OCH_3_, 4-F, 3-F, and 2-F) was transferred to a 100 mL volumetric flask, and then chromatography-grade methanol was added. The absorbance at the maximum absorption wavelength was measured using a blank methanol solution as a control, and the measurement was repeated three times. 

**Figure 5:**
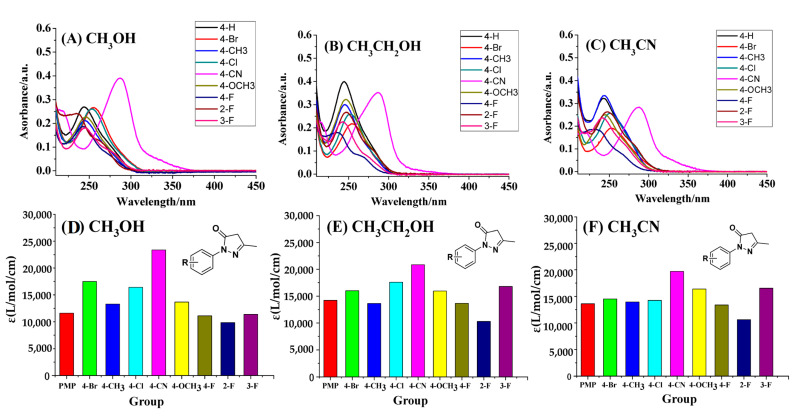
UV absorption spectra and molar extinction coefficients of PMP derivatives with different substituents when methanol (**A**,**D**), ethanol (**B**,**E**), and acetonitrile (**C**,**F**) were used as solvents.

The Lambert–Beer law describes how light absorption intensity, concentration, and light path length are related to wavelength for a given substance. In the classical equation: A = log (1/T) = εbc, ε reflects the degree of light absorption by the absorbing medium, which can be used as the characteristic constant of the substance. When b = 1, the value of ε is the slope of this binary first-order equation. The higher the ε value, the more sensitive the chemosensor will be. A Lambert–Beer law can calculate the molar extinction coefficient and absorbance, and the specific values of PMP derivatives can be found in the supplementary material. According to the above method, weighed samples were dissolved in ethanol and acetonitrile, respectively. The results of the three solvent assays are shown in Figure 5, and Tables S2–S4.

The results showed that when methanol was used as the solvent, the molar absorption coefficient of each derivative was higher, and CPMP had less crossover with other peaks at the maximum absorption wavelength. Therefore, CPMP was selected as a novel sugar chemosensor with high sensitivity and was applied to the quality control method concerning the *Polygonatum* polysaccharide used in TCM.

### 3.4. Optimizing Derivatization Condition by RSM [16]

Variables such as temperature, time, concentration of alkali, and concentration of CPMP significantly affect the efficiency of the reducing sugar-CPMP derivatization reaction; furthermore, these factors can interact. Additionally, a complete experiment designed to explore the relationships between the variables is often time-consuming. To solve this problem, the RSM was proposed to find the most valuable areas for optimization, such as reducing the difficulty of the experimental design, maximizing production, minimizing costs, and minimizing side effects. To optimize the efficiency of the derivatization of saccharides, we selected four single factors, which include reaction temperature (°C), time (h), alkali concentration (mol/L), and CPMP concentration (mol/L). The peak area of CPMP-monosaccharide was explored using Box–Behnken design (BBD)-RSM (Table 6). Based on 29 measurement experiments, we estimated the parameters of the model using the least square method (Table 2). Multiple regression analysis was utilized to analyze the experiment data, resulting in a relationship between the response variable and the test variable of a second-order equation. Using Design-Expert 11.0 software, we can compare multiple regression models and optimize process parameters based on the obtained test data. Thanks to the outstanding value of the software in comparison with alternative methods, optimal conditions were identified for verification and detection.

### 3.5. High-Performance Liquid Chromatography

This study used Agilent ZORBAX SB-C8 (4.6 mm × 250 mm, 5 μm) as a separation column with a temperature of 30 °C. Moreover, the flow rate, the injection volume, and the detection wavelength were 1 mL/min, 5 μL, and 278 nm, respectively. The mobile phase was ammonium acetate (0.02 mol/L)-acetonitrile, gradient elution (0–8 min, CH_3_CN 20%; 10–13 min, CH_3_CN 28%; 13–14 min, CH_3_CN 20%).

### 3.6. Preparation of P. cyrtonema Hua. Polysaccharides (PCPs)

The PCPs were prepared from *P. cyrtonema* powder. Briefly, the powder was soaked in boiling water (1:4 *w*/*v*, 100 °C) for 30 min and then precipitated using ethanol four times. Using Sevag’s method, the precipitates were deproteinized and lyophilized before further analysis. 

### 3.7. Monosaccharide Determination 

By modifying a previous report [16], we reacted CPMP and reducing sugar, producing the reducing sugar-CPMP derivatives. We then took 19.90 mg of CPMP, dissolved 0.2 mL DMF, and added 0.2 mL (20 mg/mL) of various standard monosaccharide solutions and the solution of 0.2 mL (0.4 mol/L) N(C_2_H_5_)_3._ This mixture was stirred and reacted at 70 °C for 60 min. We then added 0.2 mL (0.3 mol/L) hydrochloric acid and 1 mL chloroform after cooling and mixed for 30 s. The mixture was then transferred to a centrifuge tube and centrifuged (speed 2000 r/min) for 5 min; we removed the lower layer solution, added chloroform, and centrifuged again, repeating this process three times. We aspirated the supernatant and diluted it to 5.0 mL, which was then filtered through a 0.22 μm microporous membrane. An aliquot of 10 μL sample was injected into HPLC to perform the analysis.

## 4. Discussion

### 4.1. Synthesis of CPMP

PMP pre-column derivatization is the main method of quality control for polysaccharides in traditional Chinese medicine. However, the nucleophilicity is insufficient when the derivatization reagent reacts (such as PMP) with monosaccharides; additionally, its maximum absorption wavelength is 245 nm. A large proportion of compounds absorb at this wavelength, which results in a relatively large amount of background interference. To address these issues, a series of PMP derivatives with different substituents (4-H, 4-Br, 4-CH_3_, 4-Cl, 4-OCH_3_, 4-F, 3-F, and 2-F) was synthesized according to the method described above.

### 4.2. Establishment of the HPLC Method

Reaction conditions have a significant influence on the results of the reaction. Therefore, it is essential to select the reaction parameters carefully. RSM is widely used today for identifying optimal reaction conditions. The five factors selected for optimizing the efficiency of saccharide derivatization (h), reaction temperature (°C), acid type, acid concentration (eq), and molar ratio (eq) are investigated herein. It was found that the following conditions were optimal: time: 60 min, temperature: 70 °C, alkali concentration: 0.4 mol/L, and CPMP concentration: 0.6 mol/L.

The monosaccharides lack chromogenic groups, and ultraviolet absorption is very weak. The conventional HPLC-UV method cannot directly detect them. Through the derivatization reaction of CPMP and different monosaccharides under optimal conditions, the monosaccharides were labeled. Subsequently, they were separated by an Agilent ZORBAX SB-C_8_ column (4.6 mm × 250 mm, 5 μm) and were determined by a UV detector at a wavelength of 278 nm.

Next, the monosaccharides in the *P. cyrtonema* Hua. polysaccharide from Jinzhai of Anhui were examined indirectly. A PCP sample was hydrolyzed, and then a CPMP precursor was derived. As shown in Table 4, the monosaccharides of PCPs include glucose, mannose, and galactose, with a compositional ratio of 1.73:1.00:0.58. Therefore, the method is also appropriate for monosaccharide measurement in PCPs.

### 4.3. Mechanism Analysis

The possible reaction mechanism was further investigated through UV-Vis and HRMS techniques [29]. As shown in Figure 1A, the maximum absorption wavelength of CPMP is 278 nm, which is redshifted compared with that of PMP. Figure 1B shows that CPMP is attached to the benzene ring with the substituent 4-CN, increasing its molar extinction coefficient. In Figure 3A, the methanol solution of CPMP is colorless and transparent, while the solution of CPMP-Glc is orange, suggesting the absorption spectrum has changed. In the HRMS spectra of the solution of CPMP-Glc, a peak at m/z 561.2101 was observed, as depicted in Figure 3B. Based on the calculation of [2CPMP-Glc + H]^+^ is 561.2092, we deduce that the conjugated product of two CPMP and one Glc is present in the derivative system. This result further supports the structure of CPMP-Glc.

### 4.4. Comparative Analysis with Existing Methods

According to our approach, the LOD (limit of detection) for the detection of monosaccharides is less than 0.006 μg/mL. On the other hand, among the reported approaches [26,27,28], the LODs are generally higher than 0.4 mg/mL. Based on the quantum dots (QD) technique, the LOD of glucose was 0.811 μg/mL and 0.901 μg/mL, respectively. However, using the reagent TPEA-BAP, the LOD of glucose only reached 0.468 μg/mL, as shown in Table 7. In contrast, the CPMP compound gave a lower detection limit at 0.00522 μg/mL. Additionally, the CPMP reagent possesses higher sensitivity (11593 for PMP; 23,382 for CPMP) and less noise interference (detection at 245 nm for PMP and 278 nm for CPMP). As a result, our developed approach is more widely useful.

## 5. Conclusions

In this study, we have presented a chemosensor method to measure the monosaccharide composition in PCP analytes and achieved satisfactory results for establishing a method of quality control of *P. cyrtonema* Hua. This approach includes three steps: the synthesis of chemosensor CPMP, the hydrolysis of polysaccharide analyte, and the derivatization and detection of monosaccharides. Among the synthesized PMP derivatives bearing various groups, the CPMP molecule possessed higher sensitivity (ε = 23382.49 L/mol/cm in CH_3_OH) and less background interference (λ_max_ = 278 nm) and thus was chosen. According to the established HPLC approach, the main monosaccharide components of the *P. cyrtonema* Hua. polysaccharide were glucose, mannose, and galactose, with a compositional ratio of 1.73:1.00:0.58. The method is appropriate for monosaccharide analysis and content measurement and can be utilized for the quality control of the *P. cyrtonema* Hua. polysaccharide. Furthermore, the developed approach has a lower LOD (less than 0.006 μg/mL). These features give this approach significant advantages. There is a marked difference in color or transparency between CPMP and CPMP-Glc. Therefore, the adduct’s absorption spectrum is changed, providing the possibility of visualizing different monosaccharides.

## Figures and Tables

**Table 1:** The values of the molar extinction coefficient of chemosensors bearing different groups.

Different Groups	ε (L/mol/cm) CH_3_OH	ε (L/mol/cm) CH_3_CH_2_OH	ε (L/mol/cm) CH_3_CN
4-H	11,593.40	14,219.19	13,611.52
4-Br	17,489.26	16,003.25	14,516.14
4-CH_3_	13,293.05	13,634.17	13,941.38
4-Cl	16,411.64	17,587.45	14,254.44
4-CN	23,382.49	20,852.47	19,712.28
4-OCH_3_	13,663.03	15,953.65	16,379.34
4-F	11,113.26	13,659.38	13,376.55
3-F	11,371.96	16,803.28	16,541.72
2-F	9850.37	10,301.37	10,620.38

**Table 2:** Variance analysis of the BBD in derivatization of CPMP-monosaccharide.

Source	Sum of Squares	DF	Mean Square	F-Value	*p*-Value
Model	100.36	14	7.17	9.24	<0.0001
A	2.61	1	2.61	3.37	0.0878
B	23.24	1	23.24	29.95	<0.0001
C	0.1008	1	0.1008	0.1300	0.7239
D	47.20	1	47.20	60.84	<0.0001
AB	0.0225	1	0.0225	0.0290	0.8672
AC	0.2025	1	0.2025	0.2610	0.6174
AD	0.0000	1	0.0000	0.0000	1.0000
BC	0.2025	1	0.2025	0.2610	0.6174
BD	0.5625	1	0.5625	0.7250	0.4088
CD	0.4225	1	0.4225	0.5446	0.4727
A^2^	13.44	1	13.44	17.32	0.0010
B^2^	11.42	1	11.42	14.72	0.0018
C^2^	10.17	1	10.17	13.10	0.0028
D^2^	3.79	1	3.79	4.89	0.0442
Residual	10.86	14	0.7759		
Lack of Fit	3.04	10	0.3039	0.1554	0.9921
Pure Error	7.82	4	1.96		
Cor Total	111.23	28			
**R^2^ = 0.9023**	R^2^_Adj_ = 0.8047	R^2^_Pred_ = 0.7327	CV = 1.27%	Adeq Precision = 10.6553	

**Table 3:** Determination of monosaccharide composition of PCPs.

Monosaccharide	Concentration (μg/mg)	Ratio
Gal	1.88	0.58
Glc	5.62	1.73
Man	3.25	1

**Table 4:** Linearity, regression line, and LOD of the derivatives in the method.

Monosaccharides	*t*_R_ (min)	Calibration Curves	r^2^	Linear Range (μg/mL)	LOD (μg/mL)	LOQ (μg/mL)
Gal	4.78	y = 25116x + 12.52	0.9992	1-21	2.12 × 10^−3^	6.42 × 10^−3^
Glc	5.92	y = 10871x+16.178	0.9995	5-104	5.22 × 10^−3^	15.81 × 10^−3^
Man	12.03	y = 14698x + 6.1496	0.9997	3-52	5.66 × 10^−3^	17.15 × 10^−3^

**Table 5:** Precision, stability, repeatability, and accuracy of CPMP-monosaccharides.

Monosaccharides	Content in Sample (μg/mL)	Precision	Stability	Repeatability	Sample Adding Recovery
		RSD (%)	RSD (%)	RSD (%)	(%)
Gal	20	0.78	0.72	0.66	103.37
Glc	20	0.44	0.31	1.09	101.25
Man	20	0.98	1.02	1.15	99.87

**Table 6:** Levels and code of derivatization variables used in Box–Behnken design.

Variable	Coded Levels		
	−1	0	1
Temperature (A, °C)	50	70	90
Time (B, min)	30	60	90
Alkali concentration (C, mol/L)	0.3	0.4	0.5
CPMP concentration (D, mol/L)	0.4	0.6	0.8

**Table 7:** Comparison of CPMP with other glucose probes reported.

Reagent	LOD (μg/mL)	Reference
TPEA-BAP	0.468	[26]
Red-QDs@SiO_2_@green-QDs@APBA	0.811	[27]
Graphene QDs	0.901	[28]
CPMP	0.00522	This work

## Data Availability

The Supplementary Materials are available online.

## Supplementary Materials

The following supporting information can be downloaded at: https://www.mdpi.com/article/10.3390/molecules28052186/s1, Table S1: The yield optimization of CPMP- monosaccharide by Box–Behnken experimental design; Table S2: The molar absorption coefficient of PMP with different substituents in methanol solvent; Table S3: The molar absorption coefficient of PMP with different substituents in ethanol solvent; Table S4: The molar absorption coefficient of PMP with different substituents in acetonitrile solvent.

## Sample Availability

Samples of the compounds CPMP and other PMP derivatives are available from the authors.
