# Supplementary material for: A Novel Method for the Pre-Column Derivatization of Saccharides from Polygonatum cyrtonema Hua. by Integrating Lambert–Beer Law and Response Surface Methodology
Source: Molecules. 2023 Feb 26;28(5):2186. doi: 10.3390/molecules28052186 (PMC10004654; doi:10.3390/molecules28052186)
Supplement: Supplementary file 1 [file molecules-28-02186-s001.zip › molecules-2225791-supplementary.pdf]

Supporting Information

A Novel Method for the Pre-column Derivatization of Saccharides from *Polygonatum cyrtonema* Hua by Integrating Lambert-Beer Law and Response Surface Methodology

Hui Liu [1,†], Yuanyuan Zhao [1,†], Leijing Chen [1], Jiao Du [1], Hongyan Guo [2,*] and Bin Wang [1,3,*]

1 Key Laboratory of Xin'an Medicine, Ministry of Education, Anhui University of Chinese Medicine, Hefei, Anhui, 230038, China
2 Hefei Institutes of Physical Science, Chinese Academy of Sciences, Hefei, China
3 Institute of pharmaceutical chemistry, Anhui Academy of Chinese Medicine, Hefei, Anhui, 230038, China
* E-mail: hyguo@iim.ac.cn; bw5654@ahtcm.edu.cn
† These authors contributed equally to this work.

Table of Contents:

1. General Remarks **S2**
2. Box–Behnken experimental **S3**
3. UV absorbance of PMP derivatives **S4**
4. Characterization Data of Products **S5**

1. General Remarks

All substrates were purchased commercially without further purification. The yields were determined based on sulfonyl hydrazides. GC-MS spectra were measured with Bruker GC-MS 456-Scion. All new compounds were characterized by ^{1}H NMR, ^{13}C NMR and HRMS. ^{1}H and ^{13}C NMR spectra were recorded on a Bruker AC-300 FT spectrometer at 400 MHz and 100 MHz, respectively, and tetramethylsilane (TMS) was used as an internal standard. Chemical shifts (δ) and coupling constants (*J*) were expressed in ppm and Hz, respectively. All chemical shifts were reported relative to tetramethylsilane (0 ppm for ^{1}H), $CDCl_3$ (7.26 ppm for ^{1}H, 77.16 ppm for ^{13}C) and DMSO (2.50 ppm for ^{1}H, 39.52 ppm for ^{13}C), respectively. High-resolution mass spectra (HRMS) were recorded on a Waters Xevo G2-XS QTOF spectrometer (Tolerance = 10.0 ppm). All reagents were obtained from commercial sources (purity >97%) and used without further purification except for special instructions. Silica gel for column chromatography was purchased from Qingdao Haiyang Chemical Co., Ltd.

2. Box–Behnken experimental

Table S1 The yield optimization of CPMP- monosaccharide by Box–Behnken experimental design

Run	A	B	C	D	Peak area
1	50	60	0.4	0.4	71.5
2	70	60	0.5	0.8	68
3	70	60	0.4	0.6	71.9
4	90	90	0.4	0.6	71
5	50	60	0.3	0.6	68.9
6	70	30	0.4	0.4	69.8
7	50	60	0.5	0.6	67.5
8	70	90	0.5	0.6	71.1
9	50	30	0.4	0.6	66.5
10	70	60	0.4	0.6	72.08
11	70	60	0.3	0.8	67.3
12	70	60	0.4	0.6	70.15
13	70	30	0.3	0.6	67.9
14	90	60	0.4	0.8	67.8
15	70	90	0.4	0.8	68
16	70	30	0.4	0.8	66.3
17	90	60	0.5	0.6	68.9
18	70	90	0.3	0.6	70.3
19	90	30	0.4	0.6	67.8
20	70	90	0.4	0.4	73
21	70	60	0.5	0.4	70.9
22	90	60	0.4	0.4	71.9
23	90	60	0.3	0.6	69.4
24	70	60	0.4	0.6	70.19
25	50	90	0.4	0.6	69.4
26	70	30	0.5	0.6	67.8
27	70	60	0.3	0.4	71.5
28	70	60	0.4	0.6	73.45
29	50	60	0.4	0.8	67.4

3. UV absorbance of PMP derivatives with different substituents

Table S2 The molar absorption coefficient of PMP with different substituents in methanol solvent

different substituents	weight (mg)	Maximum absorbance	Maximum absorption wavelength	C（mol/L)	ε (L/mol/cm)
4-CN	5.32	0.39	287	1.67E-05	23382.49
4-CH_3	4.79	0.21	244	1.59E-05	13293.05
4-F	5.16	0.19	243	1.68E-05	11113.26
4-Cl	5.29	0.26	254	1.59E-05	16411.64
4-Br	6.14	0.27	255	1.52E-05	17489.26
4-OCH_3	5.42	0.23	246	1.66E-05	13663.03
4-H	6.46	0.27	244	2.32E-05	11593.40
2-F	7.539	0.24	234	2.45E-05	9850.37
3-F	19.55	0.18	246	1.59E-05	11371.96

Table S3 The molar absorption coefficient of PMP with different substituents in ethanol solvent

different substituents	weight (mg)	Maximum absorbance	Maximum absorption wavelength	C（mol/L)	ε (L/mol/cm)
4-CN	5.38	0.35	287	1.69E-05	20852.47
4-CH_3	6.63	0.30	244	2.20E-05	13634.17
4-F	5.09	0.23	243	1.66E-05	13659.38
4-Cl	4.9	0.26	254	1.47E-05	17587.45
4-Br	5.49	0.22	255	1.36E-05	16003.25
4-OCH_3	6.6	0.32	246	2.02E-05	15953.65
4-H	7.81	0.40	244	2.80E-05	14219.19
2-F	21.54	0.18	234	1.75E-05	10301.37
3-F	19.55	0.27	246	1.59E-05	16803.28

Table S4 The molar absorption coefficient of PMP with different substituents in acetonitrile solvent

different substituents	weight (mg)	Maximum absorbance	Maximum absorption wavelength	C（mol/L)	ε (L/mol/cm)
4-CN	4.58	0.28	287	1.44E-05	19712.28
4-CH_3	7.22	0.33	244	2.40E-05	13941.38
4-F	5.44	0.24	243	1.77E-05	13376.55
4-Cl	6.01	0.26	254	1.81E-05	14254.44
4-Br	5.31	0.19	255	1.32E-05	14516.14
4-OCH_3	5.01	0.25	246	1.53E-05	16379.34
4-H	6.59	0.32	244	2.37E-05	13611.52
2-F	21.54	0.19	234	1.75E-05	10620.38
3-F	19.55	0.26	246	1.59E-05	16541.72

4. Characterization Data of Products

1-(4-bromophenyl)-3-methyl-1H-pyrazol-5-ol. White solid: 46% yield; ^{1}H NMR (500 MHz, DMSO) δ 7.71 (d, 2H), 7.60 (d, 2H), 2.51 (s, 2H), 2.11 (s, 3H).

3-methyl-1-(p-tolyl)-1H-pyrazol-5-ol. White solid: 52% yield. ^{1}H NMR (500 MHz, DMSO) δ 7.57 (d, 2H), 7.21 (d, 2H), 2.50 (s, 2H), 2.30 (s, 3H), 2.10 (s, 3H).

1-(4-chlorophenyl)-3-methyl-1H-pyrazol-5-ol. White solid: 37% yield; ^{1}H NMR (500 MHz, DMSO) δ 7.76 (d, 2H), 7.47 (d, 2H), 2.51 (s, 2H), 2.12 (s, 3H).

1-(4-methoxyphenyl)-3-methyl-1H-pyrazol-5-ol. White solid: 41% yield; ^{1}H NMR (500 MHz, DMSO) δ 7.57 (d, 2H), 6.98 (d, 2H), 3.76 (s, 3H), 2.51 (s, 1H), 2.10 (s, 3H).

1-(4-fluorophenyl)-3-methyl-1H-pyrazol-5-ol. White solid: 76% yield; ^{1}H NMR (500 MHz, $CDCl_3$) δ 7.83 (d, 2H), 7.08 (d, 2H), 3.42 (s, 2H), 2.18 (s, 3H). ^{13}C NMR (126 MHz, $CDCl_3$) δ 170.4, 160.8, 158.9, 134.2, 120.6, 115.5, 43.0, 17.0.

1-(3-fluorophenyl)-3-methyl-1H-pyrazol-5-ol. White solid: 27% yield; ^{1}H NMR (500 MHz, $CDCl_3$) δ 7.69 (d, 2H), 7.33 (d, 1H), 6.86 (m, 1H), 3.42 (s, 2H), 2.18 (s, 3H). ^{13}C NMR (126 MHz, $CDCl_3$) δ 170.6,163.8, 161.9, 139.4, 130.1, 113.9, 111.5, 106.1, 43.2, 16.9.

1-(2-fluorophenyl)-3-methyl-1H-pyrazol-5-ol. White solid: 28% yield; ^{1}H NMR (500 MHz, $CDCl_3$) δ 7.43 (d, 1H), 7.32 (s, 1H), 7.20 (m, 1H), 7.17 (s, 1H), 3.40 (s, 2H), 2.17 (s, 3H). ^{13}C NMR (126 MHz, $CDCl_3$) δ 171.1, 156.9, 129.4, 127.1, 124.4, 116.9, 41.5, 17.1.
